# Tight Regulation of Extracellular Superoxide Points to Its Vital Role in the Physiology of the Globally Relevant *Roseobacter* Clade

**DOI:** 10.1128/mBio.02668-18

**Published:** 2019-03-12

**Authors:** Colleen M. Hansel, Julia M. Diaz, Sydney Plummer

**Affiliations:** a Department of Marine Chemistry and Geochemistry, Woods Hole Oceanographic Institution, Woods Hole, Massachusetts, USA; b Skidaway Institute of Oceanography, Department of Marine Sciences, University of Georgia, Savannah, Georgia, USA; Oregon State University

**Keywords:** *Roseobacter*, reactive oxygen species, superoxide, superoxide dismutase

## Abstract

There is a growing appreciation within animal and plant physiology that the reactive oxygen species (ROS) superoxide is not only detrimental but also essential for life. Yet, despite widespread production of extracellular superoxide by healthy bacteria and phytoplankton, this molecule remains associated with stress and death. Here, we quantify extracellular superoxide production by seven ecologically diverse bacteria within the *Roseobacter* clade and specifically target the link between extracellular superoxide and physiology for two species. We reveal for all species a strong inverse relationship between cell-normalized superoxide production rates and cell number. For exponentially growing cells of Ruegeria pomeroyi DSS-3 and *Roseobacter* sp. strain AzwK-3b, we show that superoxide levels are regulated in response to cell density through rapid modulation of gross production and not decay. Over a life cycle of batch cultures, extracellular superoxide levels are tightly regulated through a balance of both production and decay processes allowing for nearly constant levels of superoxide during active growth and minimal levels upon entering stationary phase. Further, removal of superoxide through the addition of exogenous superoxide dismutase during growth leads to significant growth inhibition. Overall, these results point to tight regulation of extracellular superoxide in representative members of the *Roseobacter* clade, consistent with a role for superoxide in growth regulation as widely acknowledged in fungal, animal, and plant physiology.

## Abstract (precis)

Formation of reactive oxygen species (ROS) through partial reduction of molecular oxygen is widely associated with stress within microbial and marine systems. Nevertheless, widespread observations of the production of the ROS superoxide by healthy and actively growing marine bacteria and phytoplankton call into question the role of superoxide in the health and physiology of marine microbes. Here, we show that superoxide is produced by several marine bacteria within the widespread and abundant *Roseobacter* clade. Superoxide levels outside the cell are controlled via a tightly regulated balance of production and decay processes in response to cell density and life stage in batch culture. Removal of extracellular superoxide leads to substantial growth inhibition. These findings point to an essential role for superoxide in the health and growth of this ubiquitous group of microbes, and likely beyond.

## INTRODUCTION

Reactive oxygen species (ROS) are short-lived oxygen radicals that form as intermediates in the reduction of oxygen to water. The ROS superoxide (O_2_^•−^), hydrogen peroxide (H_2_O_2_), and hydroxyl radical (HO^•^) are prevalent in biological and environmental systems. All aerobic organisms form ROS intracellularly as a by-product of respiration or photosynthesis. ROS, particularly superoxide and hydrogen peroxide, are also produced extracellularly by a broad range of cells, including animals, fungi, phytoplankton, and bacteria (1–9). At elevated concentrations, ROS have well-known toxic properties, including the ability to alter the redox state of critical enzymes or destroy essential biomolecules, such as membranes and proteins. For example, superoxide activates oxidative stress in macro- and microorganisms, triggers programmed cell death, fuels the ichthyotoxicity of phytoplankton blooms, and promotes the bleaching of symbiotic corals (10, 11). To maintain ROS concentrations at subtoxic levels, metabolites and enzymes that specifically target and destroy oxygen radicals (12) are tightly regulated, such as the superoxide scavenger superoxide dismutase (SOD) and hydrogen peroxide scavenger catalase.

The toxic potential of ROS has perpetuated an oversimplified view that these molecules are widely and oftentimes indiscriminately associated with stress, disease, and ultimately death. While this assumption still persists in many scientific disciplines, abundant evidence has also led to the alternative view that biological ROS production formed both intracellularly and extracellularly is beneficial in a large variety of living systems. For instance, superoxide plays a vital and long-recognized role in the immune defense response of mammalian phagocytes (13). There is also an increasing recognition that superoxide is an essential molecule required for basic cellular physiology and growth of other nonphagocytic plant and animal cell types (14, 15). In these systems, superoxide functions as a cell signal, reactant in wound repair, and autocrine growth promoter. For example, the critical role of extracellular superoxide in cell signaling, proliferation, and differentiation by fungi is well established (1). Furthermore, with the recognition of superoxide as an infochemical critical to the sensing and regulation of redox homeostasis, the last couple of decades have seen a dramatic reversal in the perception of superoxide in animal and plant physiology (14, 15).

Among microbes, evidence is also emerging in support of a role for superoxide in cell viability and proliferation by the pathogenic bacteria Escherichia coli and Salmonella enterica serotype Typhimurium, as well as the toxic raphidophyte Chattonella marina (16, 17). Within nonpathogenic microorganisms, however, the physiological purpose of extracellular superoxide production remains enigmatic. Despite its perceived role as an indicator of stress, extracellular superoxide production has been observed in healthy, actively growing cells encompassing a wide taxonomy of common marine organisms, including the diazotroph *Trichodesmium*, cyanobacterium *Synechococcus*, diatoms *Thalassiosira* spp., and various marine algae and heterotrophic bacteria (2, 4, 6, 8, 18–21). In particular, high levels of extracellular superoxide have been observed for representative members of the *Roseobacter* clade of heterotrophic alphaproteobacteria grown under ideal conditions in culture (2, 5). Here, we analyzed the dynamics and potential role of extracellular superoxide production in the baseline physiology of bacteria within the *Roseobacter* clade, a numerically abundant and geographically widespread group of microbes throughout the global ocean (22).

## RESULTS AND DISCUSSION

**FIG 1:**
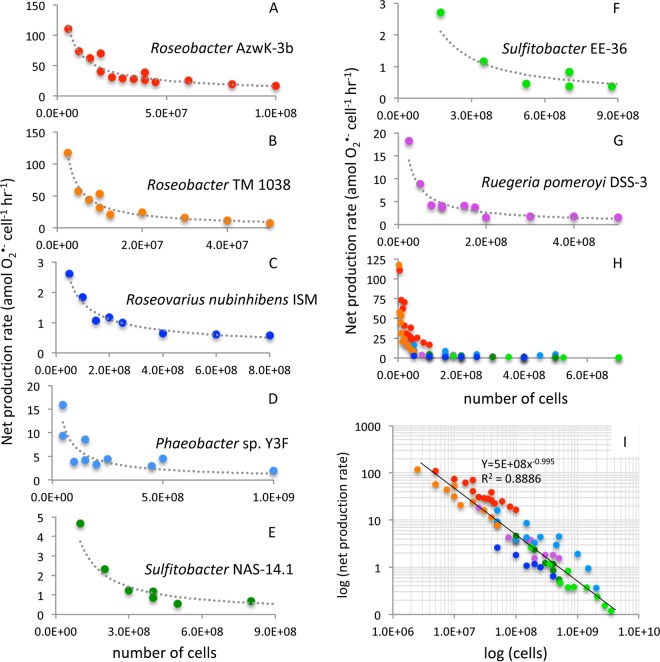
Cell-normalized extracellular superoxide production rates by seven bacterial species (A to G) within the *Roseobacter* clade. (H and I) All *Roseobacter* clade bacterial species, illustrating a strong power law relationship between cell-normalized extracellular superoxide production rates and cell number.

**FIG 2:**
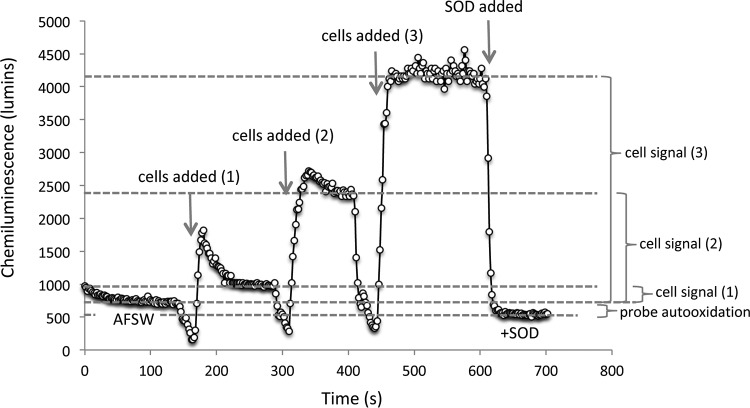
Representative chemiluminescence trace for Ruegeria pomeroyi DSS-3 at three cell loadings. The initial baseline is obtained using a carrier solution consisting of DTPA-treated, aged, filtered seawater with a filter placed in-line. Once a stable baseline is obtained, the pump is stopped, cells are added, and the pump is restarted. This process is repeated with cells added sequentially to the in-line filter. After a steady-state signal is obtained for the last loading, SOD is injected into the AFSW carrier solution to confirm that superoxide was responsible for the chemiluminescence signal. The difference between the AFSW baseline and the +SOD baseline is typically ∼200 luminescence units, and likely represents autooxidation of the MCLA probe. Thus, to obtain the most conservative cell-derived signal and avoid this autooxidation artifact, cell-derived signals are obtained by subtracting the initial AFSW signal from the signal obtained once cells are added to the in-line filter.

Extracellular superoxide was produced by seven diverse *Roseobacter* species (see Fig. S1 in the supplemental material) grown to mid-exponential phase (Fig. 1; see also Table S1 in the supplemental material). The seven strains explored here were selected to represent a wide taxonomic and ecological diversity within the *Roseobacter* clade. Extracellular superoxide was quantified via a widely used flow injection approach by pumping sterile seawater past cells hosted on an in-line filter (Fig. S2) (2, 18, 20). Superoxide was measured downstream of the filter-hosted cells via reaction with a high-sensitivity chemiluminescent probe and detected by an adjacent photomultiplier tube in real time (FeLume, Waterville Analytical) (23). Using the method described previously by Diaz et al. (2), cell-derived signals were obtained by subtracting an aged, filtered seawater baseline (with filter in-line and in the absence of SOD) from the cell-derived signal (see example chemiluminescent trace in Fig. 2 and Materials and Methods below for more detail). For all seven species, extracellular superoxide production was measured as a function of cell density by intermittently stopping flow, loading more cells onto the same in-line filter, resuming flow, and waiting for superoxide signals to stabilize. Steady-state extracellular superoxide concentrations ranged from 1.08 to 18.94 nM (mean, 5.38 nM; median, 4.03 nM) (Table S2 and Fig. S3). At low cell densities, superoxide concentrations typically increased with cell number, followed by a plateau or decrease in concentrations at higher cell numbers (Table S2 and Fig. S3). Among the seven species, cell-normalized rates of net superoxide production ranged nearly 3 orders of magnitude from 0.12 to 117.54 amol cell^−1^ h^−1^ (Fig. 1 and Table S2). Rates of extracellular superoxide production were inversely related to cell number for each species (Fig. 1).

10.1128/mBio.02668-18.1 FIG S1 Neighbor-joining phylogenetic tree based on 16S ribosomal DNA sequences of seven *Roseobacter* clade bacteria analyzed for this study. Sequences were aligned over >1,300 positions using Clustal Omega (https://www.ebi.ac.uk/tools/msa/clustalo/) and assembled into a tree using the interactive Tree of Life (Letunic and Bork, 2011; Letunic and Bork, 2007) (http://itol.embl.de/). Sequence similarity is only an estimate of evolutionary relationships. Download FIG S1, PDF file, 0.1 MB. Copyright © 2019 Hansel et al. 2019 Hansel et al. This content is distributed under the terms of the Creative Commons Attribution 4.0 International license.

10.1128/mBio.02668-18.2 FIG S2 Simplified schematic of the flow injection chemiluminescence approach used to measure extracellular superoxide in this research. Download FIG S2, PDF file, 0.2 MB. Copyright © 2019 Hansel et al. 2019 Hansel et al. This content is distributed under the terms of the Creative Commons Attribution 4.0 International license.

10.1128/mBio.02668-18.3 FIG S3 Typical steady-state superoxide concentration trends as a function of cell load in mid-exponential cultures of seven *Roseobacter* clade species. Here we illustrate the results of one loading experiment as an example of the range of trends observed. Download FIG S3, PDF file, 0.1 MB. Copyright © 2019 Hansel et al. 2019 Hansel et al. This content is distributed under the terms of the Creative Commons Attribution 4.0 International license.

10.1128/mBio.02668-18.4 TABLE S1 *Roseobacter* clade bacteria investigated in this study. Download Table S1, PDF file, 0.1 MB. Copyright © 2019 Hansel et al. 2019 Hansel et al. This content is distributed under the terms of the Creative Commons Attribution 4.0 International license.

10.1128/mBio.02668-18.5 TABLE S2 Compilation of superoxide measurements for cell loading experiments with seven *Roseobacter* species. Download Table S2, PDF file, 0.1 MB. Copyright © 2019 Hansel et al. 2019 Hansel et al. This content is distributed under the terms of the Creative Commons Attribution 4.0 International license.

**FIG 3:**
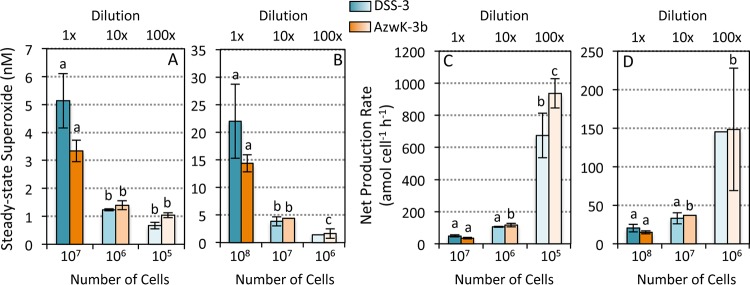
Steady-state extracellular superoxide concentrations (A and B) and cell-normalized production rates (C and D) for Ruegeria pomeroyi DSS-3 (blue) and *Roseobacter* sp. strain AzwK-3b (orange) in serially diluted cultures (0, 10, and 100× diluted) after 6 h of incubation. The same volume of culture, either 0.1 ml (A and C) or 1.0 ml (B and D) was added to the in-line filters for each measurement. Means with different letters are significantly different (*P* < 0.05); note the lack of an error bar for one treatment of DSS-3, which had an *n* of 1.

Equivalent trends in extracellular superoxide production were observed for measurements of serially diluted *Roseobacter* clade cultures. Briefly, mid-exponential-phase cells of Ruegeria pomeroyi DSS-3 and *Roseobacter* sp. strain AzwK-3b were diluted 10- and 100-fold into sterile seawater and allowed to acclimate for 6 h, and then extracellular superoxide production was analyzed by loading a single aliquot (one of two volumes—0.1 or 1.0 ml) from each dilution onto in-line filters. This approach circumvents potential stress imposed on the cells during the sequential loading processes, which involved the addition of an increasing number of cells onto the filter, combined with intermittent flow. Consistent with results from the sequential loading method (Fig. 1), superoxide levels were significantly lower and cell-normalized superoxide production rates were significantly higher in diluted cultures of both bacteria (Fig. 3). Further consistent with the inverse cell density dependence of superoxide production, cell-normalized rates within a single dilution level were higher when fewer cells (0.1 ml) were loaded (note the different vertical scales in Fig. 3C and D).

**FIG 4:**
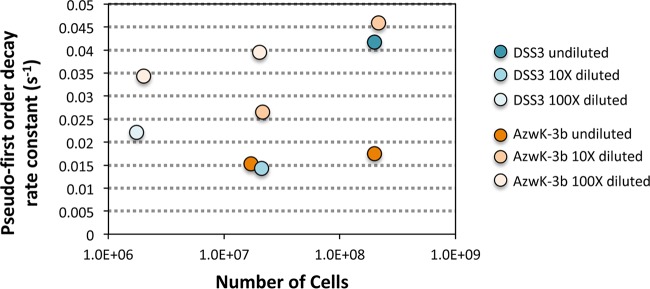
Pseudo-first-order rate constants for decay of an exogenous superoxide spike added as KO_2_ within an aged, filtered seawater matrix to Ruegeria pomeroyi DSS-3 and *Roseobacter* AzwK-3b cultures. Decay rates were obtained in undiluted and diluted (10× and 100×) cultures. Decay followed pseudo-first-order kinetics with spikes 1× to 2× the steady-state concentration measured for each culture (<15 nM).

Net superoxide production rates as a function of cell density followed an apparent power law relationship, similar to that commonly observed for cellular interactions, metabolism, evolution, and signaling behavior in numerous biological systems (Fig. 1H and I) (e.g., 24, 25). A similar inverse relationship between cell density and extracellular superoxide production has been observed in other marine microbial groups, including several harmful algae (e.g., 6, 21, 50), *Trichodesmium* (18), and a *Vibrio* bacterium (based on normalization of data in >Fig. S6 in reference 2). Furthermore, in the case of *R. pomeroyi* DSS-3 and *Roseobacter* sp. AzwK-3b, superoxide decay rates did not increase within higher-cell-number incubations despite higher superoxide concentrations and hence increased potential for catalyzed and uncatalyzed superoxide dismutation. In fact, superoxide decay did not vary systematically with cell number (Fig. 4), indicating that the cell density-driven trends in extracellular superoxide production are not a function of superoxide degradation capacity (Fig. 4). Thus, it appears that cell density-driven changes in net extracellular superoxide production are predominantly regulated by modulating gross production. Broad representation of a power law relationship between cell density and extracellular superoxide production within microbial systems points to regulated superoxide production within and beyond the *Roseobacter* clade.

**FIG 5:**
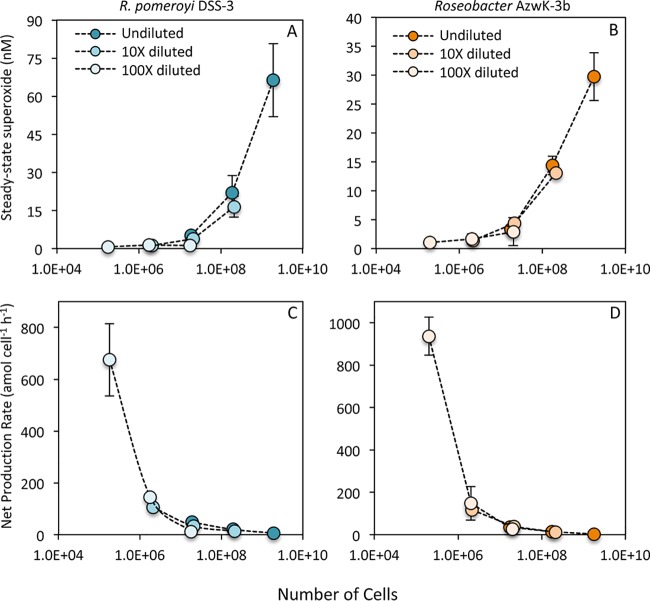
Steady-state extracellular superoxide concentrations (A and B) and cell-normalized production rates (C and D) for Ruegeria pomeroyi DSS-3 (blue) and *Roseobacter* sp. strain AzwK-3b (orange) in serially diluted cultures (undiluted and 10× and 100× diluted) at three cell loading levels following 6 h of acclimation to illustrate superoxide production as a function of cell number for each culture.

In addition to revealing the inverse cell density dependence of extracellular superoxide production, results from the sequential loading and dilution experiments also suggest that superoxide production is modulated over very short timescales. To further examine the timescale of extracellular superoxide regulation, extracellular superoxide production within the *R. pomeroyi* DSS-3 and *Roseobacter* sp. AzwK-3b dilution series was also analyzed across a range of cell loadings to provide overlapping cell numbers for each dilution level (Fig. 5). Despite the clear difference in superoxide levels for a given volume of culture (representative of a given volume within the flask), superoxide concentrations and cell-normalized production rates were not significantly different across dilution conditions when equivalent numbers of cells were added to the filter (Fig. 5). These results suggest that the 6-h preconditioning to low cell densities had no effect on the ultimate rate of extracellular superoxide production per cell, once the cells were transferred to the filter during analysis. However, the sharp exponential decline in cell-normalized production rates was again observed over increasing cell loadings (Fig. 5). These results clearly illustrate that cells respond rapidly to surrounding cell density regardless of previous incubation conditions. Thus, superoxide concentrations and cell-normalized superoxide production rates are regulated within seconds as a function of cell density, further consistent with potential involvement in cell signaling.

**FIG 6:**
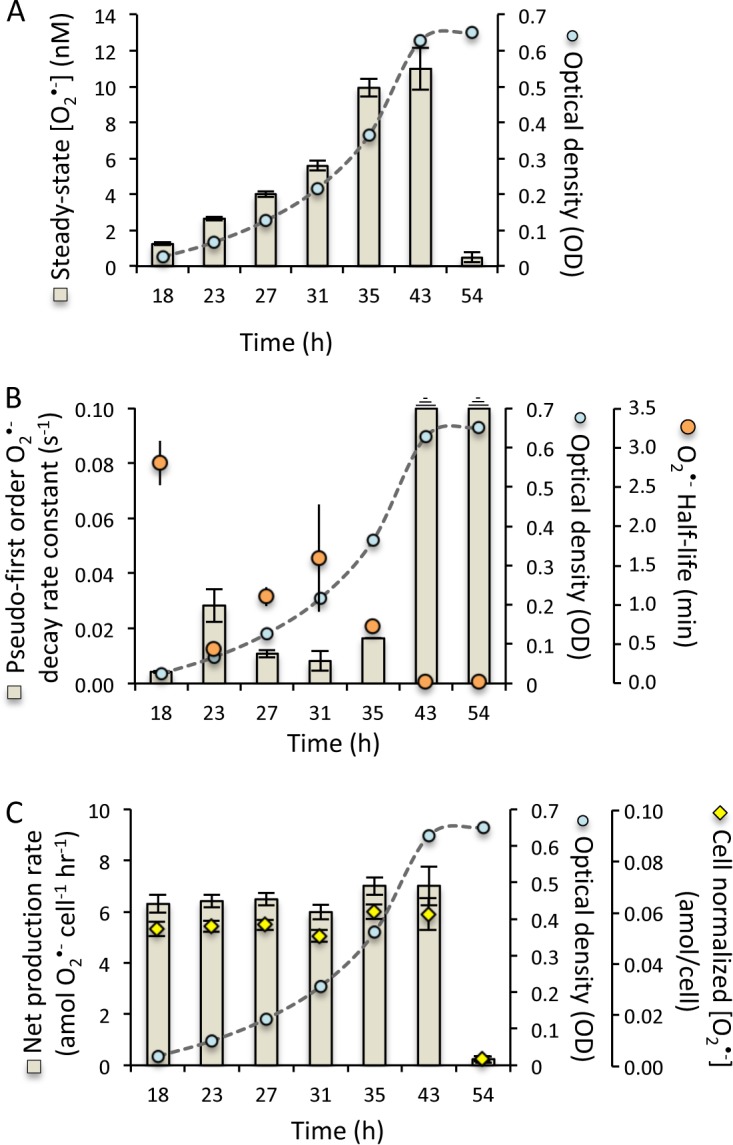
Superoxide production and decay over the life cycle of Ruegeria pomeroyi DSS-3 in batch culture. (A) Steady-state superoxide levels (in nanomolar) (tan bars) over time through a growth curve indicated by optical density (OD) at 600 nm (blue circles). (B) Pseudo-first-order rate constants for superoxide decay (seconds^−1^) (tan bars) and half-lives (in minutes) (orange circles) through a growth curve indicated by OD_600_ (blue circles). (C) Net cell-normalized superoxide production rates (in attomoles cell^−1^ hour^−1^) (tan bars) and cell-normalized superoxide concentrations (in attomoles/cell) (yellow diamonds) over a growth curve indicated by OD_600_ (blue circles).

Extracellular superoxide production is also tightly regulated during a growth cycle in batch culture through contrasting forces of production and decay. In batch cultures of *R. pomeroyi* DSS-3, extracellular superoxide concentrations steadily increased during growth but then declined sharply upon entering stationary phase (Fig. 6A). Similarly, superoxide decay increased so dramatically in stationary phase that standard additions of exogenous superoxide could not be detected (Fig. 6B; dashed lines in Fig. 6 represent rate beyond detection). By comparison, these same standard additions were readily quantified earlier in the growth curve, allowing for the calculation of decay rates and corresponding half-lives. Superoxide decay peaked during lag phase and then fluctuated throughout subsequent exponential growth (Fig. 6B). In spite of the variability in decay rates during active growth, measured rates of net superoxide production were stable throughout lag and exponential phases (Fig. 6C). These trends suggest that, in addition to decay, gross production rates were also variable during this time of active growth. Indeed, throughout this phase of dynamic production and decay, a remarkably stable cell-normalized level of superoxide is maintained outside the cell over the course of a growth curve (Fig. 6C). In contrast, cell-normalized superoxide concentrations and net production rates declined sharply in stationary phase, consistent with the observed increase in superoxide decay.

In addition to changes in cell density, the superoxide dynamics observed across the growth curve of *R. pomeroyi* DSS-3 point also to strong physiological control throughout the life cycle. For example, while superoxide concentrations increased over the growth curve (Fig. 6A), following the trend expected as cell density increases, net superoxide production rates were stable through exponential growth (Fig. 6C), deviating from the inverse cell density dependence relationship observed at a single point within the growth phase (Fig. 1, 3, and 4; mid-exponential phase). In addition, cell density did not change significantly between the last two time points in the growth curve experiment (optical density [OD] at 43 and 54 h [Fig. 6]), yet steady-state superoxide concentrations (Fig. 6A) and net superoxide production rates (Fig. 6C) did change drastically over this time frame, further underscoring a physiological explanation for the extracellular superoxide dynamics observed. Indeed, decay rates fluctuated independent of cell number throughout exponential growth, in contrast to the distinct decay rate shifts that *R. pomeroyi* DSS-3 exhibited when transitioning into stationary phase (Fig. 6B). Thus, the superoxide dynamics observed over the growth curve of *R. pomeroyi* DSS-3 show strong physiological control, which is consistent with the active maintenance of superoxide outside the cell during growth but the elimination of it once cell proliferation is no longer favorable.

The biochemical pathways responsible for extracellular superoxide production within marine bacteria, including R. pomeroyi, are not fully known but are likely enzymatically regulated. In fact, identification of the enzyme(s) responsible for extracellular superoxide production within marine bacteria is currently limited to *Roseobacter* sp. AzwK-3b, which utilizes an animal heme peroxidase (AHP) that is found within the outer membrane and cell-free exudate (26, 27). Within plants, similar peroxidases are known to oscillate between peroxidase (H_2_O_2_-degrading) and oxidase (O_2_^•−^-producing) activity that is controlled at times by extracellular hydrogen peroxide levels (28). A lack of regions encoding AHP in the genomes of the other *Roseobacter* clade organisms explored in this study points to one or more other yet to be identified enzymes or pathways responsible for superoxide production within these organisms. For example, extracellular superoxide production by E. coli and Enterococcus faecalis has been linked with electron shuttling through NADH oxidases to extracellular oxygen via quinones (29, 30). Yet in eukaryotes, transmembrane NADPH oxidases (NOX) are a well-known family of enzymes that generate extracellular superoxide. NOX homologs have been reported in mammalian cells (31), fungi (32), plants (33), seaweeds (34), and phytoplankton (4, 35, 36). NADPH oxidase has also been implicated in superoxide production within some marine microbes, including C. marina (36) and the coral algal symbiont *Symbiodinium* (37).

Regardless of the production mechanism, extracellular superoxide concentrations are ultimately offset by concomitant decay processes at the cell surface or in the external milieu, likely mediated by cell-bound or secreted enzymes and/or small molecules. Here, the activity of these unidentified decay factors was shown to fluctuate during the life cycle of *R. pomeroyi* batch cultures, ultimately completely inhibiting the buildup of extracellular superoxide during stationary phase (Fig. 6). Similar life cycle superoxide dynamics have been observed in other cell types, including microbial and animal cells. In E. coli and *C. marina* cultures, for instance, superoxide levels during active growth decline by more than 80% upon entering stationary phase (16, 38). In E. coli, this superoxide decay is controlled by a periplasmic SOD, which is expressed upon entering stationary phase only when it is secreted and attached to the outer aspect of the outer membrane (39). SOD-deficient mutants of *E. coli*, as well as *Salmonella* Typhimurium and Saccharomyces cerevisiae, grew normally during exponential phase but died on entering stationary phase, consistent with the vital role of this extracellular SOD in modulating growth (16, 17). Further, animal cell lines that exhibit cell density limitation on growth induce SOD expression at the time when cell proliferation ceases, whereas cell lines not exhibiting density limitation do not express SOD (40). These findings and others have led to the proposition that SOD is involved in growth regulation rather than simply protecting against superoxide (14, 15). Genes encoding SOD are ubiquitous throughout the *Roseobacter* clade and are likely similarly involved in superoxide regulation by these microbes.

**FIG 7:**
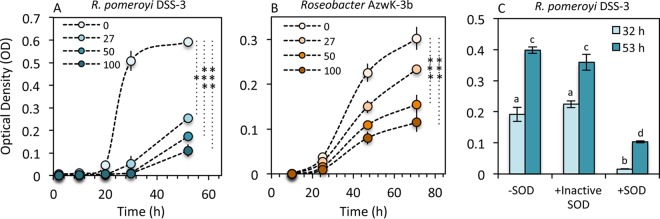
Impact of exogenous superoxide dismutase (SOD) on growth as depicted by optical density (OD) at 600 nm for Ruegeria pomeroyi DSS-3 (A) and *Roseobacter* AzwK-3b (B) at four SOD concentrations (0, 27, 50, and 100 U/ml). (C) Impact of heat-inactivated SOD on growth of Ruegeria pomeroyi DSS-3. Asterisks indicate significance levels of 0.01 to 0.05 (*), 0.001 to 0.01 (**), and 0.0001 to 0.001 (***). Means with different letters are significantly different (*P* < 0.05).

To target the potential role of extracellular superoxide production in growth regulation within the *Roseobacter* clade, we tested the response of *R. pomeroyi* DSS-3 and *Roseobacter* sp. AzwK-3b to additions of the superoxide scavenger SOD. Removal of extracellular superoxide during growth via the addition of exogenous SOD inhibited growth of both *R. pomeroyi* DSS-3 and *Roseobacter* sp. AzwK-3b in a concentration-dependent manner (Fig. 7A and B; *P* < 0.05). In *R. pomeroyi* DSS-3, these results could not be accounted for by the addition of heat-killed SOD, indicating that the growth inhibition response was specific to the active enzyme, and thus, the degradation of extracellular superoxide (Fig. 7C). These results are in line with the proposed role of superoxide in growth and are consistent with a previous study with *C. marina* that similarly observed inhibited growth in SOD-amended cultures, resulting in a lack of asexual division and a change in cell morphology (38).

Overall, direct measurements of extracellular superoxide production and decay revealed that superoxide dynamics are tightly regulated as a function of growth phase and cell density in representative members of the *Roseobacter* clade, consistent with a role for superoxide in growth regulation. These results demonstrate a physiological role for extracellular superoxide in these bacteria, as widely observed in animals, fungi, and plants (1, 41). The presence of superoxide outside the cell is thus beneficial, potentially requisite, for cell growth and/or division in representative *Roseobacter* strains. The exact mechanism of superoxide-enhanced growth is unclear, but it may involve superoxide-mediated alterations of plasma membrane lipid structure, as recently suggested for various cell types (15). Indeed, it has been suggested that a delicate balance between superoxide-generating NADPH oxidase activity and superoxide degradation by SOD controls superoxide levels at the cell surface, which ultimately modulates membrane biophysics and lipid signaling cascades (15). Alternatively or additionally, as a powerful and promiscuous redox-active molecule, superoxide could control the redox state around the cell and in particular influence the speciation and hence (bio)availability of micronutrient metals such as iron (42–44). In either case, the presence of superoxide outside the cell is clearly an intentional and beneficial process for these organisms.

A new view of the diverse and multifaceted role of superoxide in microbial health is beginning to come into focus, where intracellular and extracellular superoxide have both beneficial and detrimental influences on microbial life. The presence of superoxide and/or SOD should therefore no longer be assumed to imply oxidative stress within microbial or environmental systems. Instead, the role of superoxide in microbial physiology is clearly more nuanced, as this reactive intermediate evidently plays an essential role in microbial health, function, and growth in the ocean. With this new appreciation, further exploration should follow to target the full repertoire of physiological benefits afforded to organisms by extracellular superoxide production and the corresponding biochemical processes responsible for its biological cycling.

## MATERIALS AND METHODS

### Overview.

Axenic cultures of bacteria within the *Roseobacter* clade were grown in filter-sterilized natural seawater collected from the Vineyard Sound, Cape Cod, amended with peptone and yeast extract (K medium; pH 7.6) at 20°C on an orbital shaker (150 rpm) (45). Cells were harvested at specific points along the growth curve by monitoring optical density (600 nm) followed by quantification via flow cytometry. For a subset of incubations, cultures were diluted 10- and 100-fold in filter-sterilized Vineyard Sound seawater and incubated for 6 h. The impact of superoxide dismutase (SOD) on growth was investigated for a series of incubations by adding Cu/Zn-SOD to a final concentration of 0, 27, 50, and 100 U/ml and monitoring growth over time represented as optical density (600 nm). Bacterial extracellular superoxide production was measured on filter-hosted cells with a flowthrough FeLume Mini system (Waterville Analytical, Waterville, ME) via the specific reaction between superoxide and the chemiluminescent probe methyl *Cypridina* luciferin analog (MCLA) (Santa Cruz Biotechnology) (23) as previously conducted (2, 8, 18) and described in detail below. Bacterial superoxide decay was quantified by adding known concentrations of a calibrated potassium superoxide (KO_2_) standard to aged filtered natural seawater influent, measuring the chemiluminescent decay over time, and modeling the decay data using a pseudo-first-order decay function (2, 20). As conducted and described in detail by Diaz et al. (2), the chemiluminescent signal attributed directly to the cells was obtained by subtracting the MCLA reagent signal obtained from DTPA-treated aged filtered seawater in the absence of SOD (Fig. 2). Further, any signal artifacts that could be created by the filter were removed by conducting the baseline and the calibrations with equivalent filters in-line during analysis. Chemiluminescent signals were converted to concentration via calibration with a multipoint KO_2_ standard curve (2, 46).

### Culture preparation.

Seven bacterial species within the *Roseobacter* clade (see Fig. S1 and Table S1 in the supplemental material) that span a wide geographic and ecological diversity were grown at 20°C in 250-ml flasks containing 100 ml of K medium (2 g liter^−1^ peptone, 0.5 g liter^−1^ yeast extract, 20 mM HEPES [pH 7.6]) prepared in filter-sterilized (0.2 μm) 75% natural seawater (K-NSW) on an orbital shaker (150 rpm) (45). Triplicate cultures of each isolate were grown for 12 to 36 h with cells harvested at mid-exponential phase as determined by cell optical density at 600 nm measured using a 1-cm quartz cuvette on a Varian Cary 50 UV-Vis spectrophotometer. In a subset of experiments, subsamples of Ruegeria pomeroyi DSS-3 were collected from replicate (*n* = 3) flasks along a growth curve to measure superoxide through a life cycle. Optical density at 600 nm (OD_600_) values were converted to cell numbers via flow cytometry analysis (see below). At each sampling point, an aliquot of cells was fixed with a final concentration of 0.5% glutaraldehyde and stored at 4°C in the dark for later flow cytometry analysis.

### Culture incubations.

Mid-exponential-phase cultures of *R. pomeroyi* DSS-3 and *Roseobacter* AzwK-3b were subsampled and diluted 10× and 100× in filter-sterilized natural seawater in 250-ml flasks. Undiluted subsamples were also transferred to new sterile 250-ml flasks in equal volumes as diluted cultures to maintain consistency between cultures. The transfers were shaken at 150 rpm on an orbital shaker for 6 h and then sampled for superoxide production. Cells after incubation were fixed with a final concentration of 0.5% glutaraldehyde and stored at 4°C in the dark for later cell enumeration via flow cytometry.

A subset of incubations contained additions of superoxide dismutase (bovine) (catalog no. S7571; Sigma-Aldrich) added from a 10-kU/ml stock solution. SOD was inactivated by heating to boiling and then incubating at 100°C for 30 min. The activity of the boiled SOD was assessed by running a superoxide calibration (see below), followed by the addition of the inactivated SOD to confirm that the signal was not impacted. Active SOD was then added to confirm the superoxide signal.

### Flow cytometry.

Cell counts were conducted on a Guava EasyCyte HT flow cytometer (Millipore). Samples were diluted (1:100) with filtered seawater (0.01 µm). Samples and filtered seawater blanks were stained with SYBR Green I (Invitrogen) according to the manufacturer’s instructions and incubated in a 96-well plate in the dark at room temperature for at least 30 min. Samples were analyzed at a low flow rate (0.24 µl s^−1^) for 3 min. Bacterial cells were counted based on diagnostic forward scatter versus green fluorescence signals. Instrument-specific beads were used to calibrate the cytometer.

### Superoxide measurements.

Cells were added to 0.2-μm syringe filters and placed in-line in an FeLume Mini system (Waterville Analytical, Waterville, ME). Extracellular superoxide was measured by running aged, 0.2-μm-filtered natural seawater past the filter-supported colonies directly into the instrument where it was mixed with the superoxide-specific chemiluminescent probe methyl *Cypridina* luciferin analog (MCLA) as described in detail previously (2).

In detail, the FeLume system is composed of two separate fluid lines, one of which is dedicated to the analyte solution and the other to the MCLA reagent (Fig. S2). Both solutions are independently flushed through the system at an identical flow rate (here 3.0 ml min^−1^) using a peristaltic pump until they converge in a spiral flow cell immediately adjacent to a photomultiplier tube, which continuously acquires data that is displayed in real time using a PC interface. Similar systems have been used to generate high-sensitivity measurements of natural superoxide concentrations and decay rates (23, 46–48), as well as extracellular superoxide production by bacteria (2), phytoplankton isolates (4, 8, 20), corals and their symbionts (19, 37), and natural *Trichodesmium* colonies (18).

For calibration, primary standard solutions of potassium dioxide (KO_2_) were prepared in NaOH (pH 12.5) amended with 50 to 100 μM diethylene-triaminepentaacetic acid (DTPA) in order to sequester trace contaminants that would otherwise significantly reduce the lifetime of superoxide. Superoxide concentrations in primary standards were quantified by measuring the difference in absorbance at 240 nm before and after the addition of superoxide dismutase (SOD) (∼2 U ml^−1^) and then converting to molar units based on the molar absorptivity of superoxide corrected for the absorption of hydrogen peroxide formed during decay at the same wavelength (49). In order to create secondary standards for analysis on the FeLume, these solutions were further diluted with aged filtered natural seawater (AFSW). The seawater was filtered (0.2 μm) and amended with 50 to 75 μM DTPA to complex metals that would shorten the lifetime of ROS and then aged in the dark for 1 or 2 days to allow for complete decay of endogenous ROS. Before introducing superoxide standards to the FeLume, an in-line filter (0.22 μm; cellulose-acetate or polyethersulfone) was placed in the analyte line, where it remained throughout the duration of the experiment. (This was done to provide consistency with biological experiments [see below] and remove any potential signal artifacts created by the filter.) Next, AFSW was allowed to pass across the filter and react with the MCLA reagent (3.0 μM MCLA, 50 μM DTPA, 0.10 M MES [pH 6.0]) until a stable baseline (<4% coefficient of variation) was achieved for ∼1 min. Then, the secondary standards were pumped directly through the analyte line across the in-line syringe filter. The analyte and reagent were each pumped at a flow rate of 3.00 ± 0.05 ml min^−1^, which was confirmed gravimetrically. Because superoxide is unstable, both primary and secondary standards were used immediately after preparation.

To prepare calibration curves, the chemiluminescence signal generated from the secondary standards was baseline corrected for chemiluminescence signal arising from the autooxidation of the MCLA reagent and extrapolated back to the time at which the primary standard was diluted (*t* = 0). Baseline correction was achieved by subtracting the average background signal generated from the AFSW passing over the in-line filter, without KO_2_, and reacting with the MCLA reagent for at least 1 min, as described above. Baseline-corrected chemiluminescence data collected over several minutes of superoxide decay in standard solutions were log linear and therefore modeled using pseudo-first-order decay kinetics.

Daily calibration curves were generated from three paired observations of time zero superoxide concentration (dependent variable) and extrapolated chemiluminescence (independent variable) using linear regression. Because chemiluminescence values were baseline corrected, regression lines were forced through the origin. Calibrations yielded highly linear curves (e.g., *R*^2^ > 0.9), with sensitivities ranging from 0.6 to 3.1 chemiluminescence units per pM superoxide.

For biological experiments, as in calibration runs, a clean syringe filter (25 mm; 0.22 μm; cellulose-acetate or polyethersulfone) was placed downstream of the peristaltic pump and upstream of the flow cell in the analyte line, where it remained throughout the duration of the experiment. Stable baseline signals (<4% coefficient of variation) were generated in biological experiments from AFSW passing over the in-line filter and reacting with MCLA for at least 1 min. The pump was temporarily stopped, and cells were added to the in-line filter using a syringe to the desired cell density. Microscopic examination of the cells after analysis did not indicate visible cellular damage. The presence of cells did not alter flow rates during the experiment, as flow rates after cell addition were typically within the analytical error of the rate determined beforehand. Extracellular superoxide produced by the organisms housed on the in-line filter and released into the AFSW carrier solution was detected downstream upon mixing with the MCLA reagent in the flow cell. Chemiluminescence signals yielded in biological experiments were considered stable upon achieving a coefficient of variation (CV) equal to or less than that of the baseline for at least 1 min (CV ≤ ±4%). These signals were corrected for background chemiluminescence by subtracting the average baseline obtained immediately before the addition of cells (without SOD added) and converted to steady-state concentration measurements using the sensitivity measured in that day’s calibration (see above). The detection limit for these measurements, calculated assuming that the minimum detectable baseline-corrected signal was three times the standard deviation of the baseline, was 85 ± 19 pM. Net superoxide production rates were then calculated as the product of the steady-state superoxide concentration and flow rate (final units of picomoles hour^−1^). Production rates of superoxide by each population were normalized to the total number of cells added to provide cell-normalized rates (final units of attomoles of superoxide cells^−1^ hour^−1^) (Tables S2 and S3). Biological replicates (*n* = 3) were conducted for superoxide measurements using equivalent cell densities.

10.1128/mBio.02668-18.6 TABLE S3 Superoxide data for Ruegeria pomeroyi DSS-3 batch culture life cycle experiments. Download Table S3, PDF file, 0.1 MB. Copyright © 2019 Hansel et al. 2019 Hansel et al. This content is distributed under the terms of the Creative Commons Attribution 4.0 International license.

To calculate superoxide decay by bacteria, standard additions of superoxide were added to a subset of the cell cultures (Fig. 4). After stable chemiluminescence signals were achieved using the AFSW carrier solution, secondary standards ranging from 1.4 to 15.4 nM were prepared in an aliquot of AFSW as described above and pumped across the cells deposited onto the in-line filter. Standard additions were prepared at concentrations chosen to represent a significant (but not excessive) addition to the cell signal, a factor of typically no more than 2 times higher (median). As in calibration experiments, baseline-corrected chemiluminescence data collected over at least 1 min of decay were log linear. The signal measured immediately before the standard addition was used as the baseline. Pseudo-first-order decay rate constants (*k* in seconds^−1^) were determined by modeling the log-transformed decay data with pseudo-first-order kinetics.

To verify that the signal produced by the cells was due to superoxide, SOD (0.8 U ml^−1^) was added to the AFSW at the end of each run. SOD always caused a rapid decrease in signal to a final baseline that was typically below the initial baseline measured before cells were loaded. The difference in the initial and final baselines (∼200 chemiluminescence units) was of the same magnitude as the decrease in baseline observed when the same amount of SOD was added to the carrier solution in the absence of cells. This baseline decrease reflects either a small, yet nonzero concentration of superoxide in the AFSW carrier solutions and/or (more likely) an effect of SOD on the background chemiluminescence produced by the autooxidation of MCLA (47). To provide the most conservative value for the superoxide production rates, only the baseline produced before the cells were added (not the baseline obtained after the cell signal with SOD added) was used in biological superoxide production calculations (Fig. 2).

### Statistical analysis.

To test for differences in superoxide production and growth, a one-way analysis of variance (ANOVA) was conducted with α = 0.05. The ANOVA was followed by a Tukey test of honestly significant differences (HSD) with *P* < 0.05. Statistical tests were performed using BioVinci.
