# Supplementary material for: Tight Regulation of Extracellular Superoxide Points to Its Vital Role in the Physiology of the Globally Relevant Roseobacter Clade
Source: mBio. 2019 Mar 12;10(2):e02668-18. doi: 10.1128/mBio.02668-18 (PMC6414704; doi:10.1128/mBio.02668-18)
Supplement: FIG S1 [file mBio.02668-18-sf001.pdf]

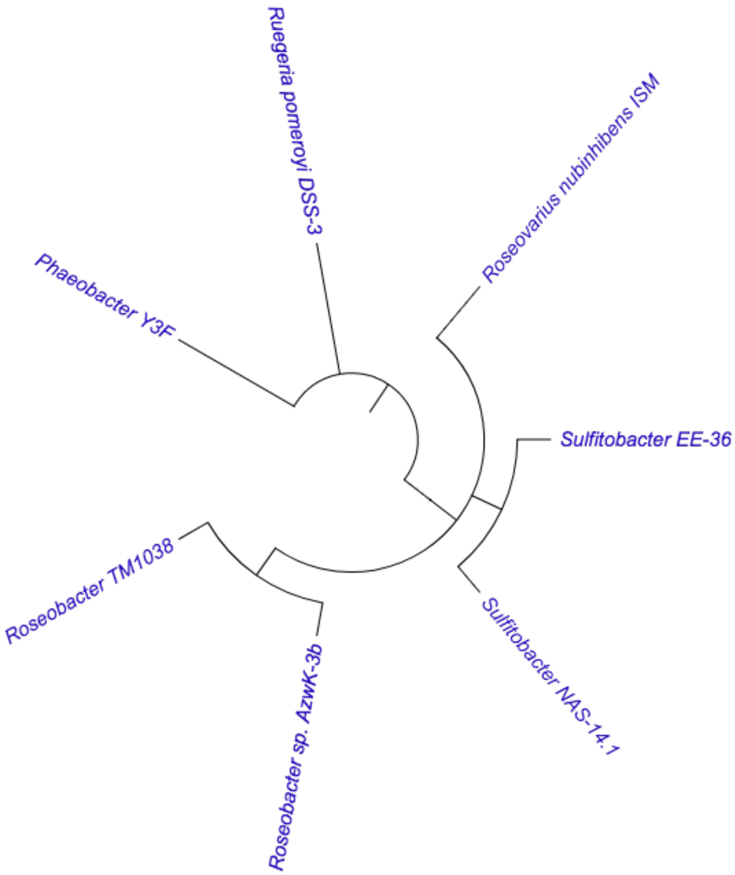

Figure S1. Neighbor-joining phylogenetic tree based on 16S ribosomal DNA sequences of seven *Roseobacter* clade bacteria analyzed for this study. Sequences were aligned over >1300 positions using Clustal Omega (https://www.ebi.ac.uk/tools/msa/clustalo/) and assembled into a tree using the interactive Tree of Life ((Letunic and Bork, 2011; Letunic and Bork, 2007); http://itol.embl.de/). Sequence similarity is only an estimate of evolutionary relationships.
