# Supplementary material for: Tight Regulation of Extracellular Superoxide Points to Its Vital Role in the Physiology of the Globally Relevant Roseobacter Clade
Source: mBio. 2019 Mar 12;10(2):e02668-18. doi: 10.1128/mBio.02668-18 (PMC6414704; doi:10.1128/mBio.02668-18)
Supplement: FIG S2 [file mBio.02668-18-sf002.pdf]

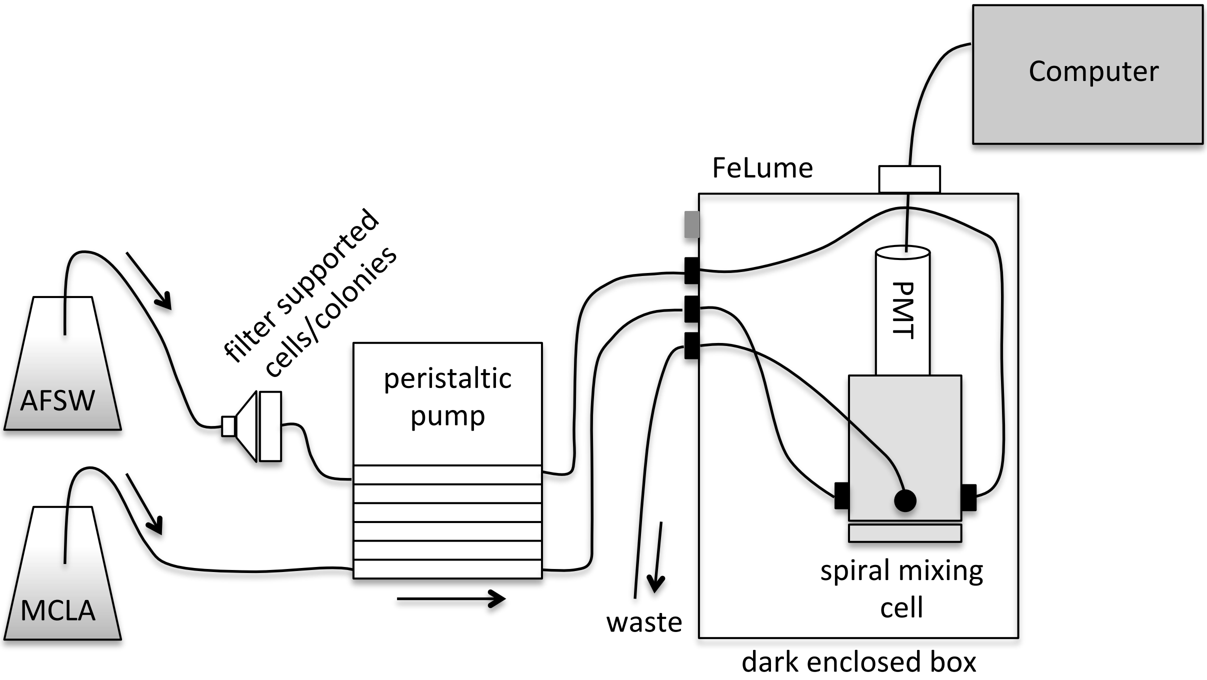

Figure S2. Simplified schematic of the flow injection chemiluminescent approach used to measure extracellular superoxide in this research.
