# Supplementary material for: Tight Regulation of Extracellular Superoxide Points to Its Vital Role in the Physiology of the Globally Relevant Roseobacter Clade
Source: mBio. 2019 Mar 12;10(2):e02668-18. doi: 10.1128/mBio.02668-18 (PMC6414704; doi:10.1128/mBio.02668-18)
Supplement: FIG S3 [file mBio.02668-18-sf003.pdf]

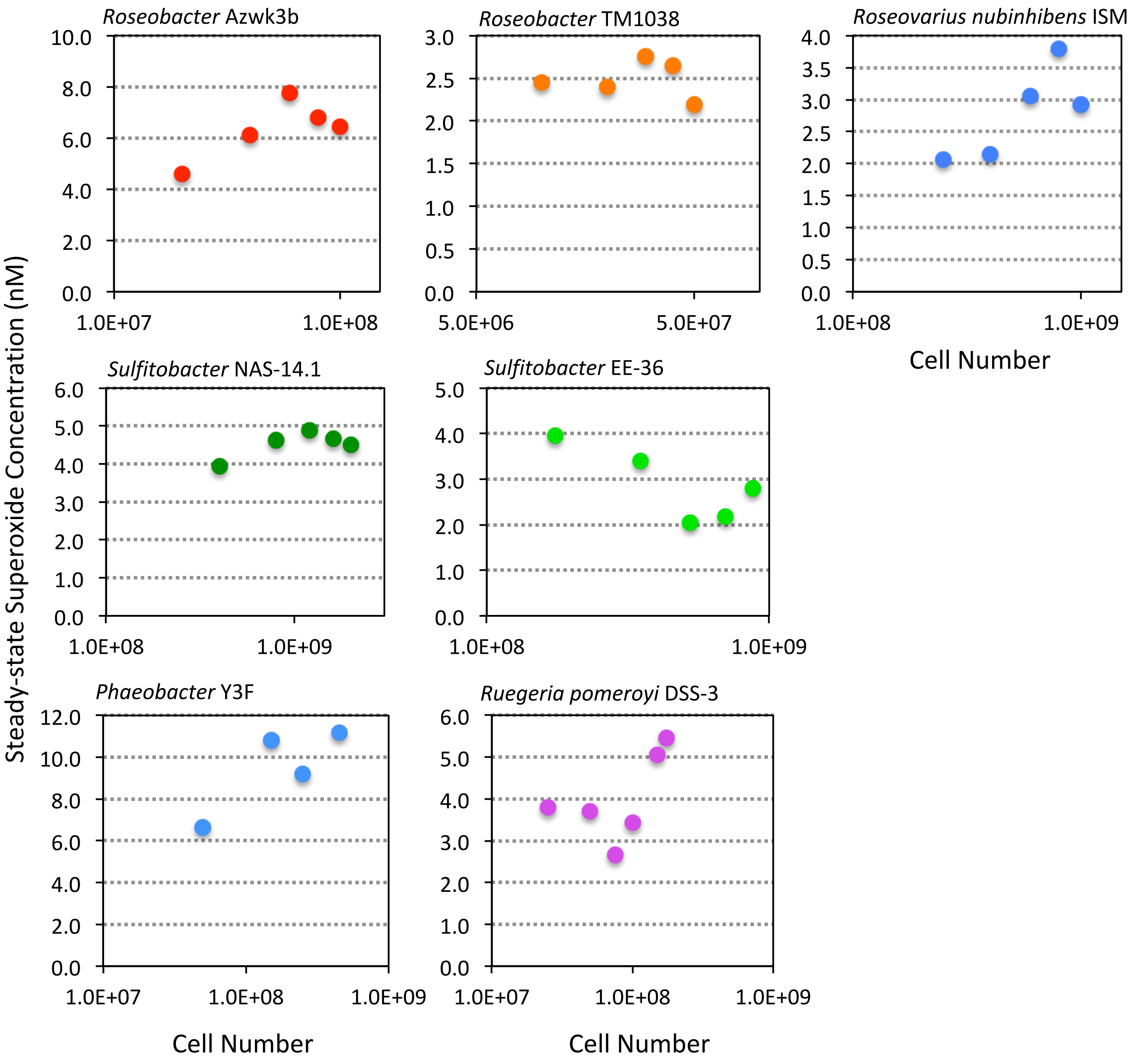

Figure S3. Typical steady-state superoxide concentration trends as a function of cell in mid-exponential cultures of 7 *Roseobacter* clade species. Here we illustrate the results of one loading experiment as an example of the range of trends observed.
