# Supplementary material for: Tight Regulation of Extracellular Superoxide Points to Its Vital Role in the Physiology of the Globally Relevant Roseobacter Clade
Source: mBio. 2019 Mar 12;10(2):e02668-18. doi: 10.1128/mBio.02668-18 (PMC6414704; doi:10.1128/mBio.02668-18)
Supplement: TABLE S1 [file mBio.02668-18-st001.pdf]

Table S1. *Roseobacter* clade bacteria investigated in this study.

Species	Isolation Location	Isolation selection conditions	Reference
Ruegeria pomeroyi DSS-3	Coastal seawater (Georgia, USA)	DMSP degradation	Gonzalez et al., 1999
Roseobacter sp. AzwK-3b	Coastal estuary water (California, USA)	Mn(II) oxidation	Francis and Hansel, 2006
Roseobacter sp. TM1038	Phycosphere of dinoflagellate *Pfiesteria piscicida*	Symbiosis; DMSP degradation	Miller and Belas, 2004
Roseovarius nubinhibens ISM	Carribean Sea surface water	non-selective	Gonzalez et al., 1999
Sulfitobacter sp. NAS-14.1	North Atlantic Ocean surface water	DMSP degradation	Moran et al., 2007
Sulfitobacter sp. EE-36	Intertidal salt marsh water (Georgia, USA)	aromatic carbon oxidation	Gonzalez et al., 1996
Phaeobacter sp. Y3F	Intertidal salt marsh water (Georgia, USA)	aromatic carbon oxidation	Gonzalez et al., 1996
