# Supplementary material for: Tight Regulation of Extracellular Superoxide Points to Its Vital Role in the Physiology of the Globally Relevant Roseobacter Clade
Source: mBio. 2019 Mar 12;10(2):e02668-18. doi: 10.1128/mBio.02668-18 (PMC6414704; doi:10.1128/mBio.02668-18)
Supplement: TABLE S2 [file mBio.02668-18-st002.pdf]

Table S2. Compilation of superoxide measurements for loading experiments with 7 *Roseobacter* species.

Organism	Cell number	Steady-state concentration (nM)	Net production rate (amol cell^{-1} h^{-1})
Roseobacter sp. AzwK-3b	2.0E+07	4.60	70.17
Roseobacter sp. AzwK-3b	4.0E+07	6.12	38.35
Roseobacter sp. AzwK-3b	6.0E+07	7.75	25.20
Roseobacter sp. AzwK-3b	8.0E+07	6.79	19.35
Roseobacter sp. AzwK-3b	1.0E+08	6.43	16.45
Roseobacter sp. AzwK-3b	5.0E+06	7.34	110.39
Roseobacter sp. AzwK-3b	1.0E+07	8.22	73.43
Roseobacter sp. AzwK-3b	1.5E+07	8.80	62.03
Roseobacter sp. AzwK-3b	2.0E+07	8.46	40.74
Roseobacter sp. AzwK-3b	2.5E+07	11.70	30.88
Roseobacter sp. AzwK-3b	3.0E+07	12.78	29.36
Roseobacter sp. AzwK-3b	3.5E+07	12.60	28.18
Roseobacter sp. AzwK-3b	4.0E+07	12.90	26.41
Roseobacter sp. AzwK-3b	4.5E+07	13.70	22.56
Ruegeria pomeroyi DSS-3	2.5E+07	3.80	18.25
Ruegeria pomeroyi DSS-3	5.0E+07	3.71	8.90
Ruegeria pomeroyi DSS-3	7.5E+07	2.66	4.26
Ruegeria pomeroyi DSS-3	1.0E+08	3.43	4.11
Ruegeria pomeroyi DSS-3	1.5E+08	5.05	4.04
Ruegeria pomeroyi DSS-3	1.8E+08	5.46	3.75
Ruegeria pomeroyi DSS-3	1.0E+08	3.20	3.84
Ruegeria pomeroyi DSS-3	2.0E+08	2.57	1.54
Ruegeria pomeroyi DSS-3	3.0E+08	4.48	1.79
Ruegeria pomeroyi DSS-3	4.0E+08	6.02	1.81
Ruegeria pomeroyi DSS-3	5.0E+08	6.44	1.55
Phaeobacter sp. Y3F	5.0E+07	6.62	15.90
Phaeobacter sp. Y3F	1.5E+08	10.80	8.64
Phaeobacter sp. Y3F	2.5E+08	9.21	4.42
Phaeobacter sp. Y3F	4.5E+08	11.17	2.98
Phaeobacter sp. Y3F	5.0E+07	3.92	9.42
Phaeobacter sp. Y3F	1.0E+08	3.23	3.87
Phaeobacter sp. Y3F	1.5E+08	5.31	4.24
Phaeobacter sp. Y3F	2.0E+08	5.54	3.32
Phaeobacter sp. Y3F	5.0E+08	18.94	4.55
Phaeobacter sp. Y3F	1.0E+09	15.88	1.91
Phaeobacter sp. Y3F	1.5E+09	12.00	0.96
Phaeobacter sp. Y3F	2.0E+09	6.07	0.36
Roseobacter sp. TM1038	1.0E+07	2.45	52.95
Roseobacter sp. TM1038	2.0E+07	2.40	24.40
Roseobacter sp. TM1038	3.0E+07	2.75	15.95
Roseobacter sp. TM1038	4.0E+07	2.65	11.53
Roseobacter sp. TM1038	5.0E+07	2.19	7.75
Roseobacter sp. TM1038	2.5E+06	4.41	117.54
Roseobacter sp. TM1038	5.0E+06	4.07	57.60
Roseobacter sp. TM1038	7.5E+06	3.99	44.07
Roseobacter sp. TM1038	1.0E+07	3.84	31.77
Roseobacter sp. TM1038	1.3E+07	3.23	21.02
Sulfitobacter sp. NAS-14.1	1.0E+08	3.89	4.67
Sulfitobacter sp. NAS-14.1	2.0E+08	3.88	2.33
Sulfitobacter sp. NAS-14.1	3.0E+08	3.06	1.22
Sulfitobacter sp. NAS-14.1	4.0E+08	2.86	0.86
Sulfitobacter sp. NAS-14.1	5.0E+08	2.30	0.55
Sulfitobacter sp. NAS-14.1	4.0E+08	3.94	1.18
Sulfitobacter sp. NAS-14.1	8.0E+08	4.63	0.69
Sulfitobacter sp. NAS-14.1	1.2E+09	4.89	0.49
Sulfitobacter sp. NAS-14.1	1.6E+09	4.67	0.35
Sulfitobacter sp. NAS-14.1	2.0E+09	4.51	0.27
Roseovarius nubinhibins ISM	5.0E+07	1.08	2.60
Roseovarius nubinhibins ISM	1.0E+08	1.53	1.83
Roseovarius nubinhibins ISM	1.5E+08	1.34	1.07
Roseovarius nubinhibins ISM	2.0E+08	1.94	1.17
Roseovarius nubinhibins ISM	2.5E+08	2.06	0.99
Roseovarius nubinhibins ISM	4.0E+08	2.14	0.64
Roseovarius nubinhibins ISM	6.0E+08	3.05	0.61
Roseovarius nubinhibins ISM	8.0E+08	3.80	0.57
Roseovarius nubinhibins ISM	1.0E+09	2.92	0.35
Sulfitobacter sp. EE-36	1.8E+08	3.95	2.71
Sulfitobacter sp. EE-36	3.5E+08	3.41	1.17
Sulfitobacter sp. EE-36	5.3E+08	2.04	0.47
Sulfitobacter sp. EE-36	7.0E+08	2.17	0.37
Sulfitobacter sp. EE-36	8.8E+08	2.80	0.38
Sulfitobacter sp. EE-36	7.0E+08	4.87	0.83
Sulfitobacter sp. EE-36	1.4E+09	4.32	0.37
Sulfitobacter sp. EE-36	2.1E+09	4.17	0.24
Sulfitobacter sp. EE-36	2.8E+09	3.65	0.16
Sulfitobacter sp. EE-36	3.5E+09	3.45	0.12
