# Supplementary material for: Tight Regulation of Extracellular Superoxide Points to Its Vital Role in the Physiology of the Globally Relevant Roseobacter Clade
Source: mBio. 2019 Mar 12;10(2):e02668-18. doi: 10.1128/mBio.02668-18 (PMC6414704; doi:10.1128/mBio.02668-18)
Supplement: TABLE S3 [file mBio.02668-18-st003.pdf]

Table S3. Superoxide data for *Ruegeria pomeroyii* DSS-3 life cycle experiments.

Timepoint	OD	Time h	Cell Number cells ml^{-1}	Mean Steady-State Concentration nM	Std Dev	Mean Cell Normalized Concentration amol/cell	Std Dev	Mean Cell Normalized Production Rate amol $cell^{-1}$ h^{-1}	Std Dev	Mean Half-life min	Std Dev	Mean Decay Rate s^{-1}	Std Dev
1	0.025	18	2.4E+07	1.245	0.067	0.053	0.003	6.31	0.34	2.76	0.28	0.004	0.000
2	0.067	23	5.0E+07	2.658	0.104	0.054	0.002	6.42	0.25	0.42	0.09	0.028	0.006
3	0.127	27	7.4E+07	4.008	0.151	0.054	0.002	6.49	0.24	1.08	0.12	0.011	0.001
4	0.216	31	1.1E+08	5.601	0.267	0.050	0.002	5.97	0.28	1.56	0.68	0.008	0.004
5	0.364	35	1.70E+08	9.934	0.492	0.059	0.003	7.00	0.35	0.70	0.00	0.017	0.000
6	0.628	43	1.9E+08	10.996	1.177	0.058	0.006	7.00	0.75	0.00	0.00	››0.1	n/a
7	0.651	54	2.5E+08	0.482	0.270	0.002	0.001	0.23	0.13	0.00	0.00	››0.1	n/a
